# Supplementary material for: The Polycomb group protein Ring1 regulates dorsoventral patterning of the mouse telencephalon
Source: Nat Commun. 2020 Nov 11;11:5709. doi: 10.1038/s41467-020-19556-5 (PMC7658352; doi:10.1038/s41467-020-19556-5)
Supplement: Supplementary file 1 — Supplementary Information [file 41467_2020_19556_MOESM1_ESM.pdf]

Supplementary Information

The Polycomb group protein Ring1 regulates dorsoventral patterning of the mouse telencephalon

Eto et al.

Supplementary Fig. 1

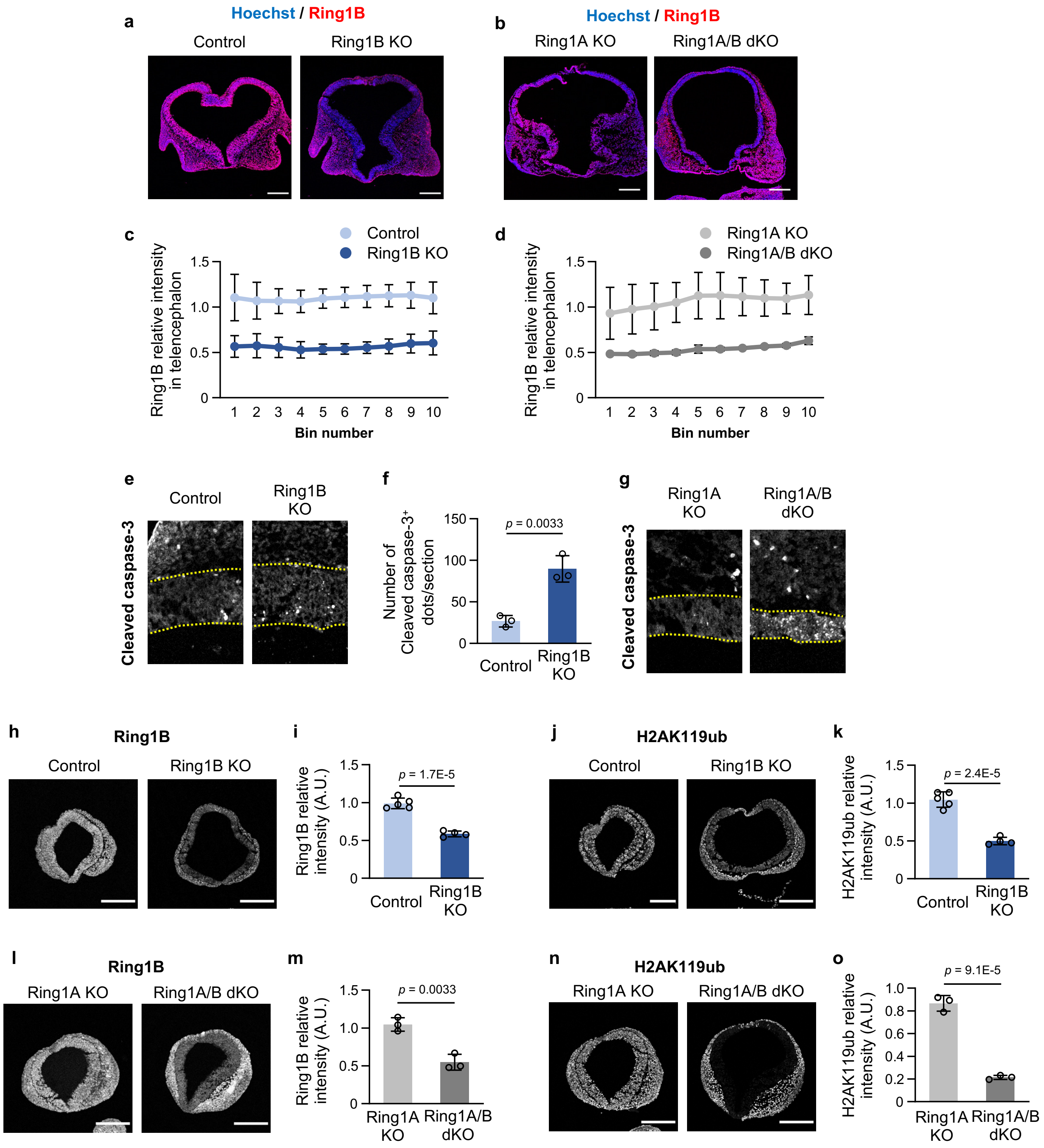

Supplementary Fig. 1 Deletion of *Ring1b* in neural tissue attenuates the expression and function of Ring1B as well as induces apoptosis in the early-stage telencephalon. a, **b, h, j, l, n** Coronal sections of the brain of control and Ring1B KO mice (**a, h, j**) or of Ring1A KO and Ring1A/B dKO mice (**b, l, n**) at E10 (**a, b**) or E9 (**h, j, l, n**) were subjected to immunohistofluorescence staining with antibodies to Ring1B (**a, b, h, l**) or H2AK119ub (**j, n**). Nuclei were counterstained with Hoechst 33342 (**a, b**). Scale bars, 200 μm. **c**, **d** Quantification of immunostaining intensity for Ring1B in images similar to those in **a** and **b**, respectively. The telencephalic wall was divided into 10 bins, from 1 (dorsal) to 10 (ventral), and the ratio of the average intensity of Ring1B in each bin to that for nonneural tissue adjacent to the ventral telencephalon was determined. Data are means ± s.d., n=3 embryos of each genotype. **e**, **g** Coronal sections of the brain of control and Ring1B KO mice (**e**) or of Ring1A KO and Ring1A/B dKO mice (**g**) at E10 were subjected to immunohistofluorescence staining with antibodies to cleaved caspase-3. A region (200 by 300 μm) of the ventral telencephalon is shown for each genotype. The telencephalic wall is demarcated by the yellow dotted lines. **f** The average number of cleaved caspase-3 signals in the control and Ring1B KO mouse telencephalon was determined for images as in **e**. Data are means ± s.d., n=3 embryos of each genotype, two-tailed Student's unpaired *t* test. **i**, **k**, **m**, **o** The ratio of the average immunostaining intensity of Ring1B (**i**, **m**) or H2AK119ub (**k**, **o**) for the entire telencephalon to that for nonneural tissue adjacent to the ventral telencephalon was determined for images similar to those in **h**, **j**, **l** and **n**, respectively. Data are means ± s.d., n=3 embryos of each genotype, two-tailed Student's unpaired *t* test. Source data are provided as a Source Data file.

Supplementary Fig. 2

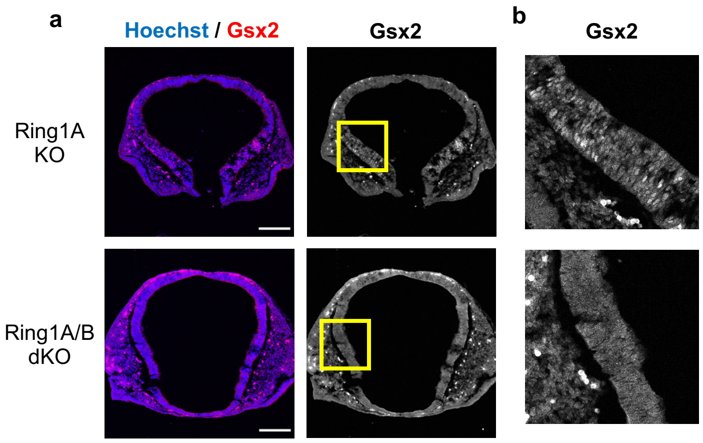

Supplementary Fig. 2 Deletion of *Ring1* attenuates expression of the LGE-specific TF Gsx2. Coronal sections of the brain of Ring1A KO or Ring1A/B dKO mice at E10 were subjected to immunohistofluorescence staining with antibodies to Gsx2. Nuclei were counterstained with Hoechst 33342. The regions indicated with yellow outlined squares (300 by 300 μm) in **a** are shown at higher magnification in **b**. Representative images from n=3 embryos of each genotype are shown. Scale bars, 200 μm.

Supplementary Fig. 3

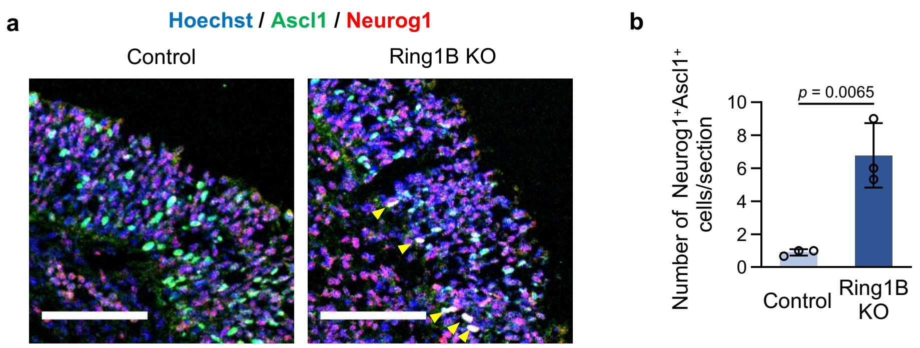

Supplementary Fig. 3. Deletion of *Ring1b* increases the number of Neurog1$^+$Ascl1$^+$ cells in the ventral telencephalon. a Higher magnification views of the regions indicated with yellow outlined squares in Fig. 3a. Yellow arrowheads indicate Neurog1$^+$Ascl1$^+$ cells. Scale bars, 100 µm. **b** Average number of Neurog1$^+$Ascl1$^+$ cells in the control and Ring1B KO mouse telencephalon determined from images similar to those in **a**. Data are means ± s.d., n=3 embryos of each genotype, two-tailed Student's unpaired *t* test. Source data are provided as a Source Data file.

Supplementary Fig. 4

a

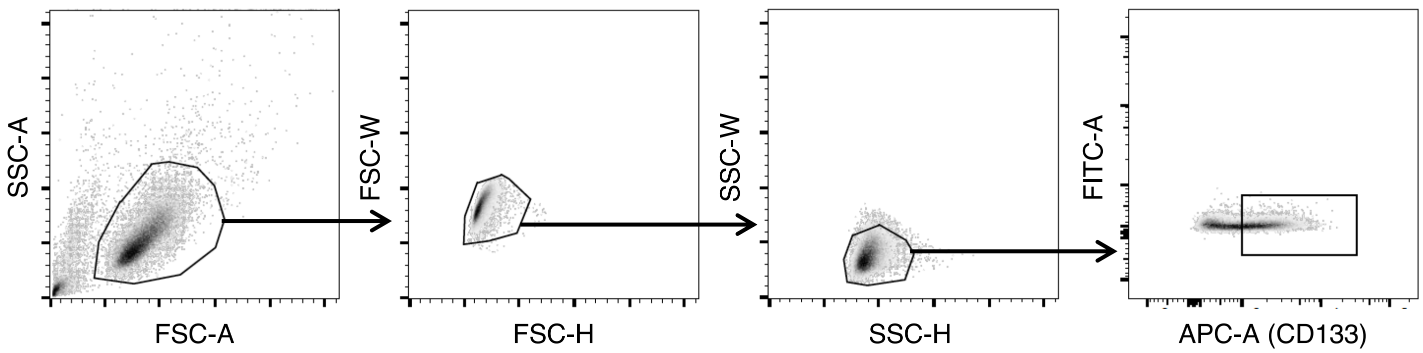

b

Up-regulated genes in Hippo signaling pathway	pvalue
Tgfb2	2.88E-11
Ppp2r2b	2.38E-08
Wnt8b	1.90E-05
Fzd1	3.88E-05
Snai2	5.43E-05
Fzd6	1.14E-04
Bmp7	1.45E-04
Bmp5	1.57E-04
Tgfbr2	3.17E-04
Tcf7	3.28E-04
Tgfb3	5.80E-04
Ctgf	1.29E-03
Wnt7b	1.51E-03
Llgl2	2.27E-03
Axin2	3.68E-03
Tead4	3.71E-03
Wnt5b	7.79E-03
Id1	1.24E-02

BMP-related

Wnt-related

c

Up-regulated BMP and Wnt ligand genes	Average RPKM Control	Average RPKM Ring1B KO	*p*value
Bmp5	2.50	10.14	1.57E-04
Bmp6	1.32	3.96	4.66E-02
Bmp7	4.38	15.54	1.45E-04
Wnt5b	20.62	37.98	7.79E-03
Wnt7b	159.45	277.73	1.51E-03
Wnt8b	14.53	41.65	1.90E-05

BMP ligand genes

Wnt ligand genes

Supplementary Fig. 4 Up-regulated genes related to the Hippo signaling pathway and fold change in BMP and Wnt ligand gene expression in ventral NPCs of Ring1B KO embryos. a Gating strategies used to sort CD133^{high} cells from dissected telecephalon at E11 on Fig. 2g, 4, 6a, b, 9 and Supplementary Fig. 8. **b** Up-regulated genes categorized to the Hippo signaling pathway for the RNA-seq analysis shown in Fig. 4 include genes related to BMP signaling (highlighted in yellow) or to Wnt signaling (highlighted in green). **c** BMP and Wnt ligand genes that showed a higher level of expression in Ring1B KO embryos than in control embryos for each of the three RNA-seq experiments shown in Fig. 4. Averages of the RPKM scores for n=3 control and Ring1B KO samples and the corresponding *p* values determined with edgeR are listed.

Supplementary Fig. 5

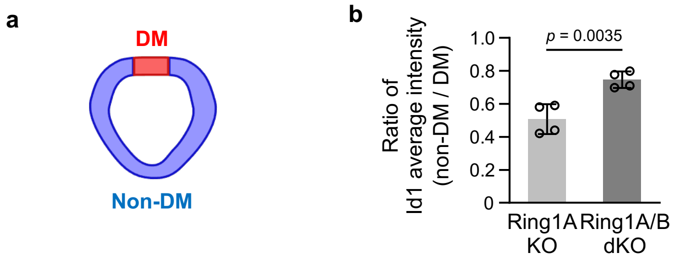

Supplementary Fig. 5 Deletion of *Ring1b* up-regulates Id1 protein expression in the telencephalon outside of the dorsal midline at E9. a Immunohistofluorescence signals of Id1 in the dorsal midline (DM) and the non-DM portion of the neuroepithelium were measured in coronal sections of the Ring1A KO and Ring1A/B dKO telencephalon at E9. **b** Ratio of Id1 average intensity in the non-DM region to that in DM. Data are means ± s.d., n=4 mice of each genotype from three litters, two-tailed Student's unpaired *t* test. Source data are provided as a Source Data file.

Supplementary Fig. 6

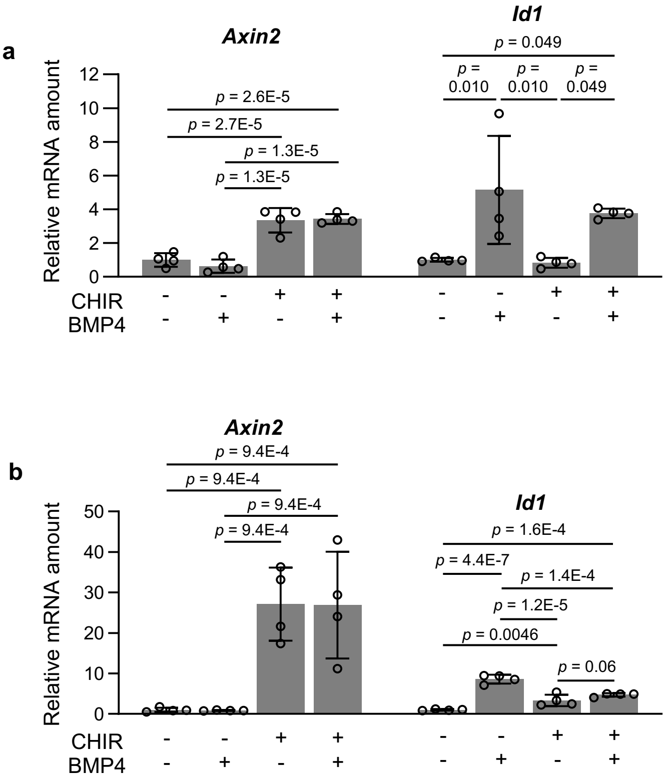

Supplementary Fig. 6 Effects of Wnt and BMP signaling on *Axin2* and *Id1* expression in cultured NPCs of the early-stage telencephalon. The telencephalon of WT (ICR) mice at E9 was maintained in explant (**a**) or monolayer (**b**) culture for 6 h before exposure for an additional 24 h to BMP4 (50 ng/ml) or 5 μM CHIR-99021. Relative *Axin2* or *Id1* mRNA abundance (normalized by the amount of *Actb* mRNA) was then determined by RT-qPCR analysis. Data are means ± s.d., n=4 independent experiments, one-way ANOVA followed by the Benjamini-Hochberg multiple-comparison test. Source data are provided as a Source Data file.

Supplementary Fig. 7

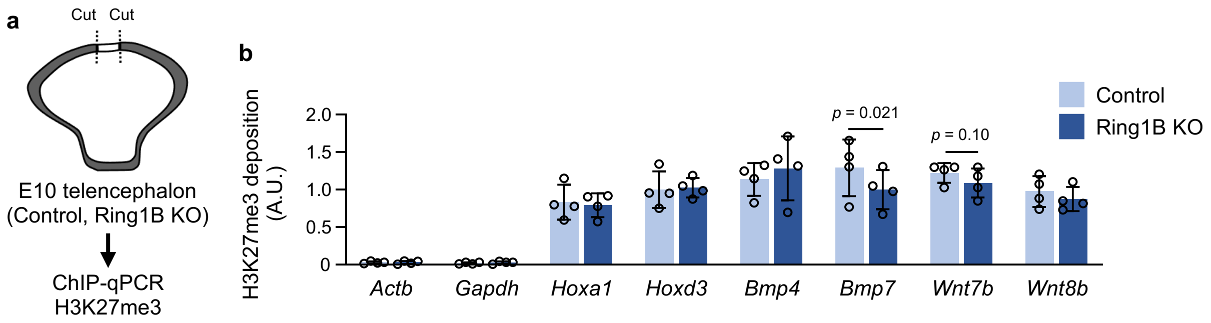

Supplementary Fig. 7 Effects of *Ring1b* deletion on H3K27me3 deposition at BMP and Wnt ligand genes in the early-stage telencephalon. a The telencephalon (outside of the dorsal midline) was isolated from control and Ring1B KO mice at E10 for ChIP-qPCR analysis with antibodies to H3K27me3. **b** ChIP-qPCR analysis of H3K27me3 deposition at the indicated gene promoters as in **a**. Data are expressed as percentage input normalized by the average value for the *Hoxa1* promoter in control mice, means ± s.d., n=4 independent experiments with embryos from three litters, two-tailed Student's paired *t* test. Source data are provided as a Source Data file.

Supplementary Fig. 8

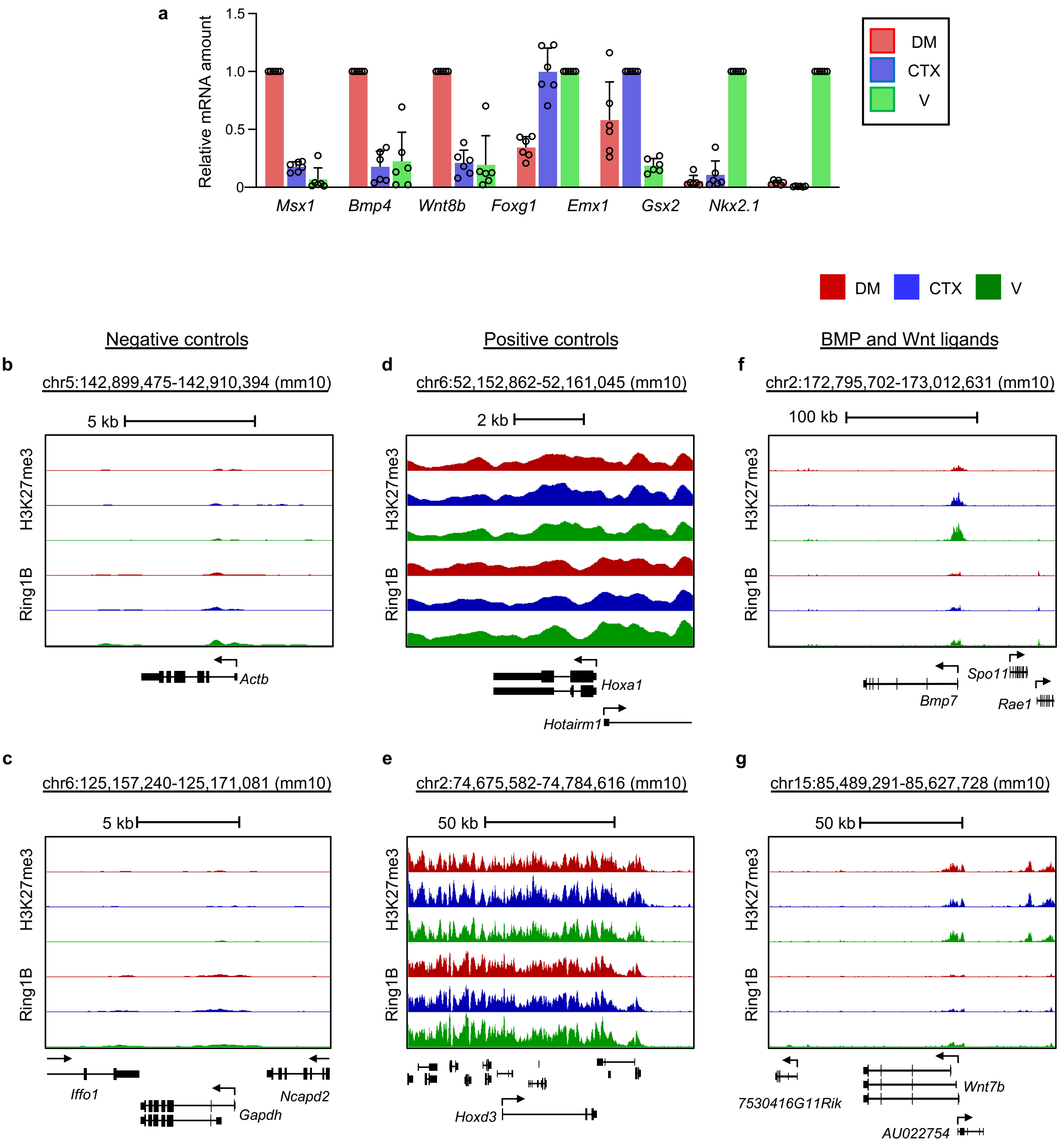

Supplementary Fig. 8 CUT&Tag analysis of H3K27me3 deposition and Ring1B binding at gene loci in NPCs of telencephalic regions along the D-V axis. a Confirmation of dissected telencephalic regions by analysis of regional marker gene expression. After dissection of the telencephalon at E11 into the dorsal midline (DM), CTX, and ventral (V) regions, $CD133^{+}$ NPCs were isolated as in Figure 9 and subjected to RT-qPCR analysis of the relative abundance of *Msx1*, *Bmp4*, and *Wnt8b* (DM markers); *Foxg1* (CTX and V marker); *Emx1* (CTX marker); and *Gsx2* and *Nkx2.1* (V markers) mRNAs (normalized by the amount of *Actb* mRNA). Data are means + s.d., n=6 independent experiments. **b**–**e** Positive and negative control loci for the CUT&Tag analysis performed in Fig. 9. The averages of normalized CUT&Tag signals for H3K27me3 and Ring1B around *Actb* (**b**) or *Gapdh* (**c**) as negative controls and around *Hoxa1* (**d**) or *Hoxd3* (**e**) as positive controls were determined for three (H3K27me3) or four (Ring1B) independent experiments and are visualized in the UCSC genome browser. The RefSeq gene models are shown at the bottom of each panel. **f**, **g**, The averages of normalized CUT&Tag signals for H3K27me3 and Ring1B around *Bmp7* (**f**) or *Wnt7b* (**g**) were determined for three (H3K27me3) or four (Ring1B) independent experiments and are visualized in the UCSC genome browser. The RefSeq gene models are shown at the bottom of each panel. Source data are provided as a Source Data file.

Supplementary Fig. 9

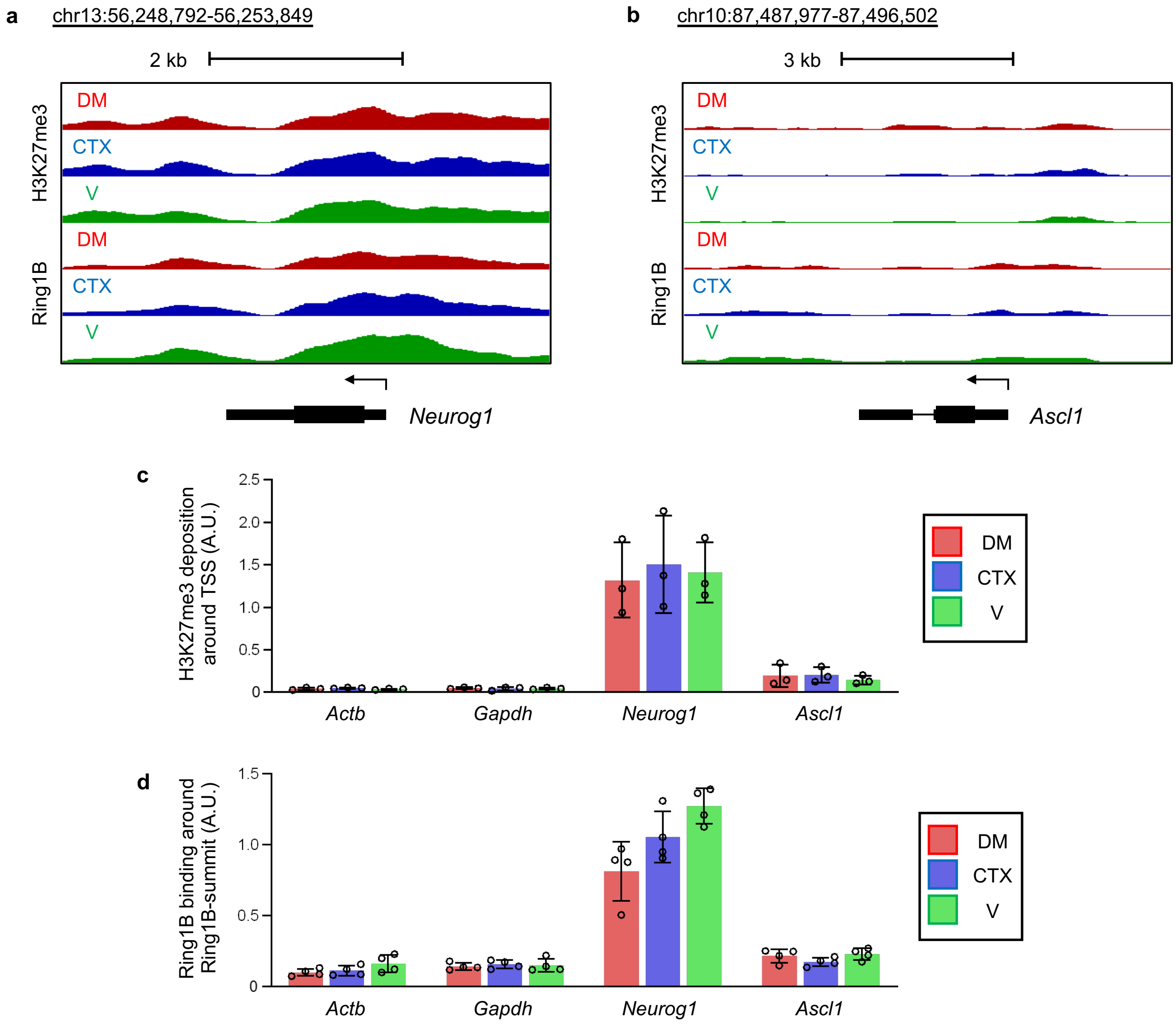

Supplementary Fig. 9 CUT&Tag analysis of H3K27me3 deposition and Ring1B binding at *Neurog1* and *Ascl1* loci. a, b The averages of normalized CUT&Tag signals for H3K27me3 and Ring1B around *Neurog1* (**a**) or *Ascl1* (**b**) were determined for three (H3K27me3) or four (Ring1B) independent experiments and are visualized in the UCSC genome browser. The RefSeq gene models are shown at the bottom of each panel. **c, d** Averages of normalized CUT&Tag signals for H3K27me3 around (±1 kbp) the transcription start site (TSS) (**c**) and Ring1B around (±1 kbp) the Ring1B summit (determined as the site in the gene body with the highest Ring1B signals across all samples) (**d**) of the indicated genes. Data are means ± s.d. for biological triplicates (**c**) or quadruplicates (**d**). Source data are provided as a Source Data file.

Supplementary Fig. 9

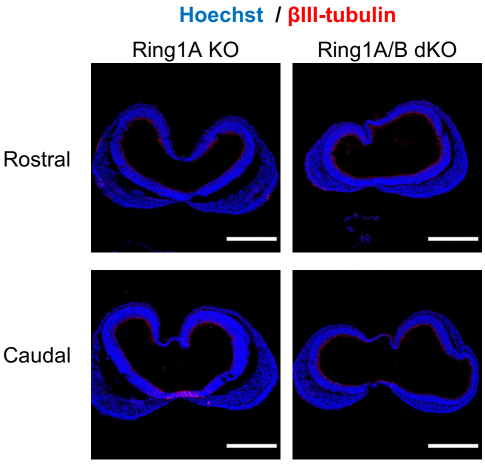

Supplementary Fig. 10 Deletion of *Ring1b* in neural tissue with *Sox1-Cre* does not promote the onset of neurogenesis in the telencephalon. Coronal sections of the brain of Ring1A KO or Ring1A/B dKO mice at E9 were subjected to immunohistofluorescence staining with antibodies to βIII-tubulin. Nuclei were counterstained with Hoechst 33342. Representative images from n=3 embryos of each genotype are shown. Scale bars, 200 µm. Note that a small number of cells were positive for the neuronal marker βIII-tubulin in the Ring1A KO telencephalon—in particular, in the ventrocaudal part—but that *Ring1b* deletion did not increase the number of these cells.

Supplementary Fig. 10

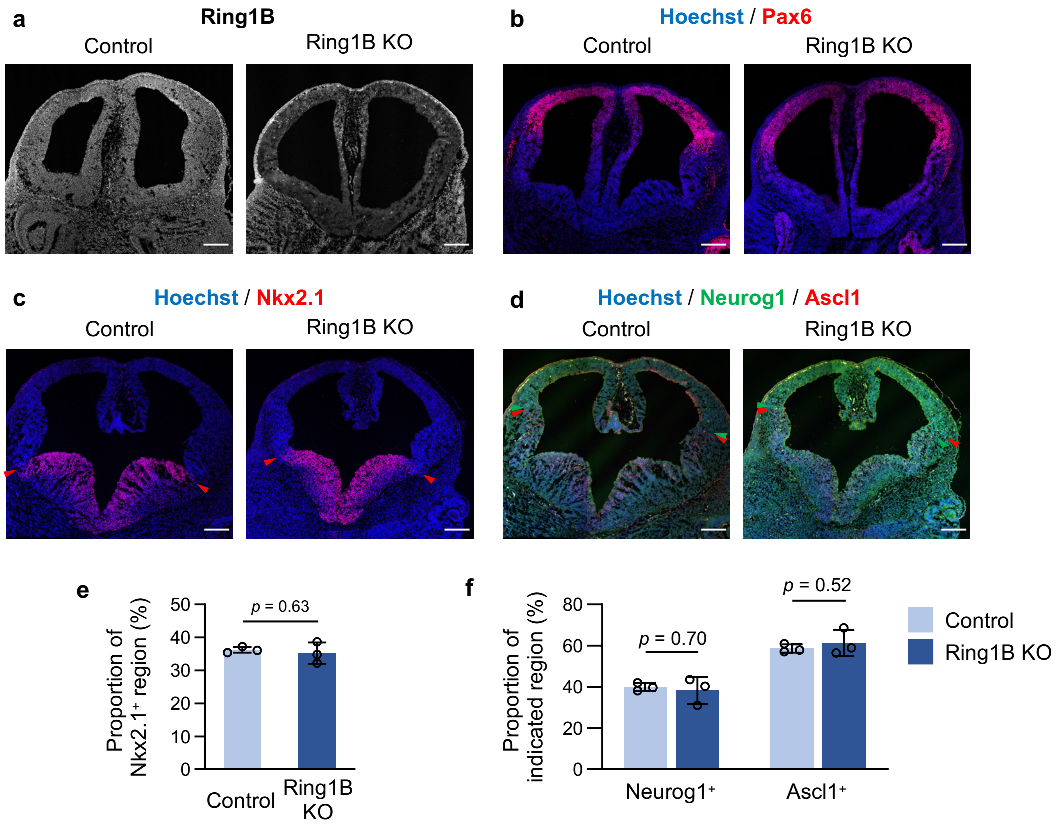

Supplementary Fig. 11 Deletion of *Ring1b* in the telencephalon with *Foxg1-IRES-Cre* does not confer the dorsal-like expression pattern of region-specific TFs in the ventral telencephalon. a–d Coronal sections of the brain of control (*Ring1b*$^{\text{flox/flox}}$ or *Ring1b*$^{\text{flox/+}}$) or Ring1B KO (*Ring1b*$^{\text{flox/flox}}$;*Foxg1-IRES-Cre*) mice at E11 were subjected to immunohistofluorescence staining with antibodies to Ring1B (**a**), to Pax6 (**b**), to Nkx2.1 (**c**), or to Neurog1 and Ascl1 (**d**). Nuclei were counterstained with Hoechst 33342 in **b** through **d**. Scale bars, 200 µm. Red arrowheads in **c** indicate the dorsal border of the Nkx2.1$^+$ region. Green and red arrowheads in **d** represent the dorsal and ventral borders of Ascl1$^+$ and Neurog1$^+$ regions, respectively. **e**, **f**, Quantification of immunostaining in images similar to those in **c** and **d**, respectively. The perimeter length of Nkx2.1$^+$ (**e**) or of Neurog1$^+$ or Ascl1$^+$ (**f**) regions as a proportion of total perimeter length for the telencephalic wall was determined. Data are means ± s.d., n=3 embryos of each genotype. Note that the proportion of the dorsal (Neurog1$^+$) and ventral (Nkx2.1$^+$ and Ascl1$^+$) telencephalic regions did not differ significantly (two-tailed Student's unpaired *t* test) between these Ring1B-deficient mice and control mice at E11. Source data are provided as a Source Data file.

Supplementary Table 1. Primer sequences for RT-qPCR

Gene	Forward primer	Reverse primer
β-actin	AATAGTCATTCCAAGTATCCATGAAA	GCGACCATCCTCCTCTTAG
Nkx2.1	GATGAGTCCAAAGCACACG	CTCCATGCCCACTTTCTTGTA
Gsx2	ACATACCTAAACCTGTCAGAGA	CTTGCAGCTTGTGTGATTG
Emx1	GTTCCCAGAGGCCATGA	TGGCCAAAGAAGCGATT
Bmp4	CTGAACTGAGTGCCATTTCC	CTCTACCACCATCTCCTGATAA
Bmp7	CAGACACTGGTTCACTTCAT	TTAGAGCTGTCGTCGAAGTAG
Id1	TACGACATGAACGGCTGCTA	TCTCCACCTTGCTCACTTT
Wnt7b	ACCAAAACTTGCTGGACCAC	ACGTGTTGCACTTGACGAAG
Wnt8b	CGTTCTTCTAGTCACTTGTGT	GGTCCCAAGCAAACTGGTATTTA
Axin2	GGTTCCGGCTATGTCTTT	CTCTCTCTGGAGCTGTT
Ptch1	TAAGAGTTTCAGCAATGTGAAGTATG	TGAAGCCAGTCTCTAAAGTAGT
Gli1	TGCCTGGAGAGACACAAT	TGGGCACCTCATGTAGC
Shh	ATGTGTTCCGTTACCAGCGA	ATATAACCTTGCCTGCCGCT
Msx1	CTACGCAAGCACAAGACCAA	GCAATAGACAGGTACTGCTTC
Foxg1	TCCCTCTACTGGCCCAT	GTGGTCCCGTTGTAACTCAAA

Supplementary Table 2. Primer sequences for ChIP-qPCR

Gene	Forward primer	Reverse primer
β-actin	CGGTTTGGACAAAGACCC	AAAGCCGTATTAGGTCCATC
Gapdh	TGCAGTCCGTATTTATAGGAACC	CTTGAGCTAGGACTGGATAAGCA
Hoxa1	CTGAACTGGCAAGAGGTGA	CGACCACGCAGAGATTT
Hoxd3	ACCTATTTGCGGTCGTC	CAAATTCGCCTGGGAAATCA
Bmp4	TGCTGCCCAAACTGATG	TTCAACGTTTGGGAATCCT
Bmp7	CAGCCTTCACCCAGAATG	CACCCTAGGACTTCAAGAG
Wnt7b	GGCATCCAGGAGTCAGA	CGCTATGGATGGAGCTAC
Wnt8b	ACTGTTTGGGATCGCTTAC	ACAAGTGACTAGAAGAACGC

Supplementary Table 3. A sequence for *Shh* ISH probe

Gene	Probe target sequence
Shh	NM_009170.3, 339-1652 (CDS 1-1314)

Supplementary Table 4. Antibodies and molecular probes for Immunohistofluorescence

Antigen	Host	Manufacturer	Serial No.	Concentration
Ascl1 (Mash1)	Mouse	BD Pharmagen	556604	1:500
Neurog1	Goat	Santa Cruz	sc-19231	1:200
H2AK119ub	Rabbit	Cell Signaling Technology	8240S	1:1000
Ring1B	Rabbit	Cell Signaling Technology	5694S	1:200
Ring1B	Mouse	MBL	D139-3	1:500
Cleaved Caspase-3	Rabbit	Cell Signaling Technology	9664S	1:1000
Nkx2.1 (TTF1)	Rabbit	Abcam	ab76013	1:1000
Gsx2 (Gsh2)	Rabbit	Millipore	ABN162	1:200
Pax6	Rabbit	Millipore	AB2237	1:500
Id1	Rabbit	Biocheck	BCH-1/37-2	1:200
βIII-tubulin	Mouse	BioLegend	801202	1:1000
Anti-goat IgG (H+L) antibody, Alexa Fluor 633	Donkey	Thermo Fisher Scientific	A-21082	1:1000
Anti-mouse IgG (H+L) antibody, Alexa Fluor 546	Donkey	Thermo Fisher Scientific	A10036	1:1000
Anti-mouse IgG (H+L) antibody, Alexa Fluor 555	Donkey	Thermo Fisher Scientific	A-31750	1:1000
Anti-mouse IgG (H+L) antibody, Alexa Fluor 594	Donkey	Thermo Fisher Scientific	A-21203	1:1000
Anti-mouse IgG (H+L) antibody, Alexa Fluor 647	Donkey	Thermo Fisher Scientific	A-31571	1:1000
Anti-mouse IgG (H+L) antibody, Alexa Fluor 647	Chicken	Thermo Fisher Scientific	A-21463	1:1000
Anti-rabbit IgG (H+L) antibody, Alexa Fluor 488	Donkey	Thermo Fisher Scientific	A-21206	1:1000
Anti-rabbit IgG (H+L) antibody, Alexa Fluor 555	Donkey	Thermo Fisher Scientific	A-31572	1:1000
Anti-rabbit IgG (H+L) antibody, Alexa Fluor 594	Donkey	Thermo Fisher Scientific	A-21207	1:1000
Anti-rabbit IgG (H+L) antibody, Alexa Fluor 647	Donkey	Thermo Fisher Scientific	A-31573	1:1000
Hoechst 33342		Molecular Probes		

Supplementary Table 5. An antibody for FACS

Antigen	Host	Manufacturer	Serial No.	Concentration
CD133-APC	Rat	Biolegend	141208	1:400

Supplementary Table 6. Antibodies for ChIP

Antigen	Host	Manufacturer	Serial No.	Concentration
H3K27me3	Mouse	MBL	MABI0323	2 µg/sample
Ring1B	Rabbit	Cell Signaling Technology	5694S	3 µg/sample

Supplementary Table 7. Antibodies for CUT&Tag

Antigen	Host	Manufacturer	Serial No.	Concentration
H3K27me3	rabbit	Cell Signaling Technology	9733S	1 µg/sample
Ring1B	rabbit	Cell Signaling Technology	5694S	1 µg/sample
Anti-rabbit IgG (H&L) antibody	Guinea pig	Rockland	611-201-122	1 µg/sample
