# Supplementary material for: The Polycomb group protein Ring1 regulates dorsoventral patterning of the mouse telencephalon
Source: Nat Commun. 2020 Nov 11;11:5709. doi: 10.1038/s41467-020-19556-5 (PMC7658352; doi:10.1038/s41467-020-19556-5)
Supplement: Supplementary file 3 — Reporting Summary [file 41467_2020_19556_MOESM3_ESM.pdf]

nature research

Corresponding author(s): Yusuke Kishi

Last updated by author(s): Sep 2, 2020

Reporting Summary

Nature Research wishes to improve the reproducibility of the work that we publish. This form provides structure for consistency and transparency in reporting. For further information on Nature Research policies, see our Editorial Policies and the Editorial Policy Checklist.

Statistics

For all statistical analyses, confirm that the following items are present in the figure legend, table legend, main text, or Methods section.

n/a	Confirmed	
☐	☒	The exact sample size (*n*) for each experimental group/condition, given as a discrete number and unit of measurement
☐	☒	A statement on whether measurements were taken from distinct samples or whether the same sample was measured repeatedly
☐	☒	The statistical test(s) used AND whether they are one- or two-sided<br>*Only common tests should be described solely by name; describe more complex techniques in the Methods section.*
☒	☐	A description of all covariates tested
☐	☒	A description of any assumptions or corrections, such as tests of normality and adjustment for multiple comparisons
☐	☒	A full description of the statistical parameters including central tendency (e.g. means) or other basic estimates (e.g. regression coefficient) AND variation (e.g. standard deviation) or associated estimates of uncertainty (e.g. confidence intervals)
☐	☒	For null hypothesis testing, the test statistic (e.g. *F*, *t*, *r*) with confidence intervals, effect sizes, degrees of freedom and *P* value noted<br>*Give P values as exact values whenever suitable.*
☒	☐	For Bayesian analysis, information on the choice of priors and Markov chain Monte Carlo settings
☒	☐	For hierarchical and complex designs, identification of the appropriate level for tests and full reporting of outcomes
☒	☐	Estimates of effect sizes (e.g. Cohen's *d*, Pearson's *r*), indicating how they were calculated

Our web collection on statistics for biologists contains articles on many of the points above.

Software and code

Policy information about availability of computer code

Data collection	Images were taken by Leica SP5 confocal microscope (Leica), Axiovert 200M microscope (camera : Axiocam or Axiocam 305) (Zeiss) or SMZ18 (camera : DS-Ri2) (Nicon).<br>qPCR analyses were performed by LightCycler 480 or LC96 (Roche).<br>Sequencing analyses were performed by Hiseq2500 Novaseq6000 or HiseqX (Illumina).
Data analysis	ELAND v2, Bowtie2, Deeptools v3.3.1 and edgeR softwares were used for sequencing analyses.<br>ImageJ and Excel (Microsoft) were used for image analyses.

For manuscripts utilizing custom algorithms or software that are central to the research but not yet described in published literature, software must be made available to editors and reviewers. We strongly encourage code deposition in a community repository (e.g. GitHub). See the Nature Research guidelines for submitting code & software for further information.

Data

Policy information about availability of data

All manuscripts must include a data availability statement. This statement should provide the following information, where applicable:

- Accession codes, unique identifiers, or web links for publicly available datasets
- A list of figures that have associated raw data
- A description of any restrictions on data availability

The data that support this study are available from the corresponding author upon reasonable request. The sequence data have been deposited in the DNA Data Bank of Japan (DDBJ) Sequence Read Archive under the following IDs: DRA008366 [https://ddbj.nig.ac.jp/DRASearch/submission?acc=DRA008366] (Quartz-seq analysis for NPCs of Ring1B KO embryos), DRA010033 [https://ddbj.nig.ac.jp/DRASearch/submission?acc=DRA010033] (CUT&Tag analysis of H3K27me3 for NPCs from DM, CTX, and V regions of the mouse telencephalon), and DRA010296 [https://ddbj.nig.ac.jp/DRASearch/submission?acc=DRA010296] (CUT&Tag analysis of

Ring1B for NPCs from DM, CTX, and V regions of the mouse telencephalon). WIG files for CUT&Tag analysis were also deposited in the DDBJ Genomic Expression Archive under the following ID: E-GEAD-378 [ftp://ftp.ddbj.nig.ac.jp/ddbj_database/gea/experiment/E-GEAD-000/].

Field-specific reporting

Please select the one below that is the best fit for your research. If you are not sure, read the appropriate sections before making your selection.

☒ Life sciences ☐ Behavioural & social sciences ☐ Ecological, evolutionary & environmental sciences

For a reference copy of the document with all sections, see nature.com/documents/nr-reporting-summary-flat.pdf

Life sciences study design

All studies must disclose on these points even when the disclosure is negative.

Sample size	Sample size was predetermined based on literature data using the same well established experimental approaches in similar animal models in our laboratory (Hirabayashi et al,, Neuron, 2009; Morimoto-Suzki et al., Development, 2015; Tsuboi et al., Dev. Cell, 2018).
Data exclusions	No data exclusions.
Replication	We confirm that all experiments in this study were replicated successfully at least three times in three different mice or in three independent cell cultures. The same experimental protocol was followed by identical steps of data analysis. Figure legends and Source Data contain exact number of samples and animals used in this study.
Randomization	All sample allocation were randomized.
Blinding	All data were analyzed under blinded conditions.

Reporting for specific materials, systems and methods

We require information from authors about some types of materials, experimental systems and methods used in many studies. Here, indicate whether each material, system or method listed is relevant to your study. If you are not sure if a list item applies to your research, read the appropriate section before selecting a response.

Materials & experimental systems

n/a	Involved in the study	
☐	☒	Antibodies
☐	☒	Eukaryotic cell lines
☒	☐	Palaeontology and archaeology
☐	☒	Animals and other organisms
☒	☐	Human research participants
☒	☐	Clinical data
☒	☐	Dual use research of concern

Methods

n/a	Involved in the study	
☒	☐	ChIP-seq
☒	☐	Flow cytometry
☒	☐	MRI-based neuroimaging

Antibodies

Antibodies used

Ascl1 mouse BD Pharmagen 556604
H3K27me3 mouse MBL MABI0323
H3K27me3 rabbit Cell Signaling Technology 9733S
Neurog1 goat Santa Cruz sc-19231
H2AK119ub rabbit Cell Signaling Technology 8240S
Ring1B rabbit Cell Signaling Technology 5694S
Ring1B mouse MBL D139-3
Cleaved Caspase-3 rabbit Cell Signaling Technology 9664S
Nkx2.1 rabbit Abcam ab76013
Gsx2 rabbit Millipore ABN162
Pax6 rabbit Millipore AB2237
Id1 rabbit Biocheck BCH-1/37-2
βIII-tubulin mouse BioLegend 801202
Anti-rabbit IgG (H&L) antibody guinea pig Rockland 611-201-122
CD133-APC rat Biolegend 141208
Donkey anti-Goat IgG (H+L) Cross-Adsorbed Secondary Antibody, Alexa Fluor 633 Thermo Fisher Scientific A-21082
Donkey anti-Mouse IgG (H+L) Highly Cross-Adsorbed Secondary Antibody, Alexa Fluor 546 Thermo Fisher Scientific A-10036
Donkey anti-Mouse IgG (H+L) Highly Cross-Adsorbed Secondary Antibody, Alexa Fluor 555 Thermo Fisher Scientific A-31750

Donkey anti-Mouse IgG (H+L) Highly Cross-Adsorbed Secondary Antibody, Alexa Fluor 594 Thermo Fisher Scientific A-21203
Donkey anti-Mouse IgG (H+L) Highly Cross-Adsorbed Secondary Antibody, Alexa Fluor 647 Thermo Fisher Scientific A-31571
Chicken anti-Mouse IgG (H+L) Cross-Adsorbed Secondary Antibody, Alexa Fluor 647 Thermo Fisher Scientific A-21463
Donkey anti-Rabbit IgG (H+L) Highly Cross-Adsorbed Secondary Antibody, Alexa Fluor 488 Thermo Fisher Scientific A-21206
Donkey anti-Rabbit IgG (H+L) Highly Cross-Adsorbed Secondary Antibody, Alexa Fluor 555 Thermo Fisher Scientific A-31572
Donkey anti-Rabbit IgG (H+L) Highly Cross-Adsorbed Secondary Antibody, Alexa Fluor 594 Thermo Fisher Scientific A-21207
Donkey anti-Rabbit IgG (H+L) Highly Cross-Adsorbed Secondary Antibody, Alexa Fluor 647 Thermo Fisher Scientific A-31573

Validation

https://www.bdbiosciences.com/us/applications/research/stem-cell-research/ectoderm-markers/mouse/purified-mouse-anti-mash1-24b72d111/p/556604
https://www.funakoshi.co.jp/contents/137110
https://www.cellsignal.com/products/primary-antibodies/tri-methyl-histone-h3-lys27-c36b11-rabbit-mab/9733
https://www.scbt.com/ja/p/neurogenin-1-antibody-a-20
https://www.cellsignal.com/products/primary-antibodies/ubiquityl-histone-h2a-lys119-d27c4-xp-rabbit-mab/8240
https://www.cellsignal.com/products/primary-antibodies/ring1b-d22f2-xp-rabbit-mab/5694
https://ruo.mbl.co.jp/bio/e/dtl/A/?pcd=D139-3
https://www.cellsignal.com/products/primary-antibodies/cleaved-caspase-3-asp175-5a1e-rabbit-mab/9664
https://www.citeab.com/antibodies/757408-ab76013-anti-ttf1-antibody-ep1584y
https://www.merckmillipore.com/JP/ja/product/Anti-Gsh2-Antibody,MM_NF-ABN162
https://www.merckmillipore.com/JP/ja/product/Anti-PAX6-Antibody,MM_NF-AB2237
http://www.biocheckinc.com/inserts/bch1_37-2.pdf
https://www.biolegend.com/en-us/products/purified-anti-tubulin-beta-3-tubb3-antibody-11580
https://rockland-inc.com/store/Whole-IgG-Affinity-Purified-Secondary-Antibodies-611-201-122-O4L_11801.aspx
https://www.biolegend.com/en-us/products/apc-anti-mouse-cd133-antibody-7243

Eukaryotic cell lines

Policy information about cell lines

Cell line source(s)	HEK293T
Authentication	The authors declare that the cell line was authenticated.
Mycoplasma contamination	The cell line was tested negative for mycoplasma contamination.
Commonly misidentified lines (See ICLAC register)	*Name any commonly misidentified cell lines used in the study and provide a rationale for their use.*

Animals and other organisms

Policy information about studies involving animals; ARRIVE guidelines recommended for reporting animal research

Laboratory animals	Jcl:ICR (CLEA Japan), Slc:ICR (SLC Japan), C57BL/6J. All mice used for mating were2 months of age ore more. Both male and female embryos were used in our study.
Wild animals	No study involving wild animals.
Field-collected samples	No study collected from the field.
Ethics oversight	All animals were maintained and studied according to protocols approved by the Animal Care and Use Committee of The University of Tokyo.

Note that full information on the approval of the study protocol must also be provided in the manuscript.
