# Supplementary material for: The Polycomb group protein Ring1 regulates dorsoventral patterning of the mouse telencephalon
Source: Nat Commun. 2020 Nov 11;11:5709. doi: 10.1038/s41467-020-19556-5 (PMC7658352; doi:10.1038/s41467-020-19556-5)
Supplement: Supplementary file 5 — Description of Additional Supplementary Files [file 41467_2020_19556_MOESM5_ESM.pdf]

Title: Supplementary Data 1

Description: The full list of differentially expressed genes and enriched pathways. The up-regulated and down-regulated genes (FDR <0.15, determined with edgeR) and up-regulated and down-regulated KEGG pathways (Benjamini score <0.15, determined with DAVID Bioinformatics Resources) defined in the analysis of genome-wide gene expression in NPCs isolated from the GE of control or Ring1B KO mice at E11 in Fig. 4 are now listed in the sheets of “Up-regulated genes”, “Down-regulated genes”, “KEGG_up-regulated” and “KEGG_down-regulated”, respectively. Refseq_ID, Gene symbol, *p* value and FDR of each determined gene are provided in the sheets of “Up-regulated genes” and “Down-regulated genes”. Pathway name, *p* value and Benjamini score of each enriched pathway are provided in the sheets of “KEGG_up-regulated” and “KEGG_down-regulated
